# Identification of a distinct population of CD133^+^CXCR4^+^ cancer stem cells in ovarian cancer

**DOI:** 10.1038/srep10357

**Published:** 2015-05-28

**Authors:** Michele Cioffi, Crescenzo D’Alterio, Rosalba Camerlingo, Virginia Tirino, Claudia Consales, Anna Riccio, Caterina Ieranò, Sabrina Chiara Cecere, Nunzia Simona Losito, Stefano Greggi, Sandro Pignata, Giuseppe Pirozzi, Stefania Scala

**Affiliations:** 1 Molecular Immunology and Immuneregulation, Istituto Nazionale per lo Studio e la Cura dei Tumori "Fondazione Giovanni Pascale" IRCCS-ITALIA. Via Mariano Semmola 80131, Naples, Italy.; 2 Stem Cell Unit, Istituto Nazionale per lo Studio e la Cura dei Tumori "Fondazione Giovanni Pascale" IRCCS-ITALIA. Via Mariano Semmola 80131, Naples, Italy.; 3 Uro-Gynecological Oncology, Istituto Nazionale per lo Studio e la Cura dei Tumori "Fondazione Giovanni Pascale" IRCCS-ITALIA. Via Mariano Semmola 80131, Naples, Italy.; 4 Pathology; Istituto Nazionale per lo Studio e la Cura dei Tumori “Fondazione Giovanni Pascale” IRCCS-ITALIA. Via Mariano Semmola 80131, Naples, Italy

## Abstract

CD133 and CXCR4 were evaluated in the NCI-60 cell lines to identify cancer stem cell rich populations. Screening revealed that, ovarian OVCAR-3, -4 and -5 and colon cancer HT-29, HCT-116 and SW620 over expressed both proteins. We aimed to isolate cells with stem cell features sorting the cells expressing CXCR4^+^CD133^+^ within ovarian cancer cell lines. The sorted population CD133^+^CXCR4^+^ demonstrated the highest efficiency in sphere formation in OVCAR-3, OVCAR-4 and OVCAR-5 cells. Moreover OCT4, SOX2, KLF4 and NANOG were highly expressed in CD133^+^CXCR4^+^ sorted OVCAR-5 cells. Most strikingly CXCR4^+^CD133^+^ sorted OVCAR-5 and -4 cells formed the highest number of tumors when inoculated in nude mice compared to CD133^−^CXCR4^−^, CD133^+^CXCR4^−^, CD133^−^CXCR4^+^ cells. CXCR4^+^CD133^+^ OVCAR-5 cells were resistant to cisplatin, overexpressed the ABCG2 surface drug transporter and migrated toward the CXCR4 ligand, CXCL12. Moreover, when human ovarian cancer cells were isolated from 37 primary ovarian cancer, an extremely variable level of CXCR4 and CD133 expression was detected. Thus, in human ovarian cancer cells CXCR4 and CD133 expression identified a discrete population with stem cell properties that regulated tumor development and chemo resistance. This cell population represents a potential therapeutic target.

According to the cancer stem cell hypothesis^1^, like adult tissues, tumors arise from cells that exhibit the ability to self-renew by asymmetric cell division. Cancer stem cells (CSC) are able to generate tumors in secondary recipients^2^ since they retain the essential property of self-protection through the activity of multiple drug resistance transporters. Acquired drug resistance may develop in initially responding tumors through selection of intrinsically resistant cells^3^. These cells have innate drug resistance by virtue of their capacity to remain quiescent^4^. CSC have frequently been isolated using specific markers for normal stem cells of the same organ; in particular CD24 (ligand for P-selectin), CD44 (hyaluronan receptor), CD133, EpCAM (epithelial cell adhesion molecule) have been used to fractionate CSCs in several solid tumors together with some functional assays as side population with ABC transporter and aldehyde dehydrogenase activity^5^. With the intent to target cell populations with innate drug resistance and potential metastatic activity, the concomitant expression of CXCR4 and CD133 was evaluated in the NCI 60 tumor cell line panel comprising cell lines derived from hematopoietic malignancies and several solid tumors (lung cancer, central nervous system (CNS), colon, breast, ovarian, and prostate cancer and melanoma) extensively characterized for patterns of gene expression^6^,^7^.

CD133 is the human homologue of mouse Prominin-1, a five transmembrane glycoprotein domain and a cell surface protein originally found on neuroepithelial stem cells in mice^8^. CD133 has been used to identify normal and cancer stem cells from several different tissues, such as hematopoietic^9^ or leukemia^10^, neural^11^ or brain tumour cells^12^, renal epithelial^13^ or kidney cancer^14^ cells and pancreatic cancer^15^. The stromal cell-derived factor-1 (SDF-1) or CXCL12/CXCR4 axis, critical for the trafficking/homing of hematopoietic stem cells^16^, was reported in adult stem cells, such as neural^17^, liver^18^, skeletal muscle satellite cells^19^, NSCLC^20^, renal^21^ and prostate^22^. CXCR4 expression on hematopoietic precursors regulates the physiological interactions with stromal bone marrow cells producing CXCL12. The most clinically advanced CXCR4 antagonist, plerixafor, is approved as an hematopoietic stem cells mobilizing agent^23^. However, the expression of CXCR4 on leukemic cells allows binding to the CXCL12 produced by marrow stromal cells, and segregates leukemic cells in bone marrow niche where they evade chemotherapy^24^. Previous evidence has demonstrated a CXCR4 functional axis in prostate and pancreatic cancer progenitors^25^,^15^. In pancreatic cancer concomitant expression of CD133 and CXCR4 identified a specific population of migrating cancer stem cells capable of evading the primary tumor and reaching distant sites. In primary non small cell lung cancer CD133^+^, epithelial specific population, is increased compared with normal lung tissue and has higher tumorigenic potential in SCID mice^26^.

The aim of the study was to evaluate two putative cancer stem cell markers, CD133 and CXCR4, in the NCI 60 cell lines to identify a cancer stem cell rich population as *in vitro* models and suggestive for translational studies in patients.

## Results

### CXCR4 and CD133 protein levels in the NCI 60 Cell Lines

**Figure 1:**
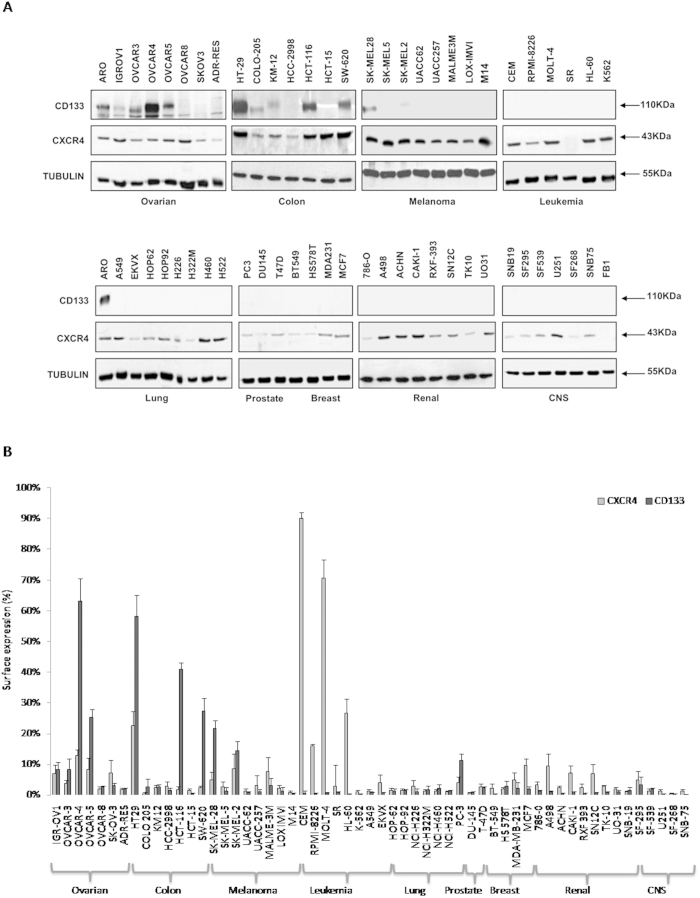
CXCR4 and CD133 protein expression in NCI 60 cell lines. ** (A)** CXCR4 and CD133 protein level was evaluated through Immunoblotting. All gels had been run under the same experimental conditions. **(B)** Bar plots summarizing Flow Cytometry analysis for CXCR4 and CD133 protein level in the 60 cell lines from the Drug Screen Program.

CXCR4 and CD133 RNA expression for the NCI 60 cell lines was available on the DCTP website (www.dtp.nci.nih.gov). To evaluate the corresponding protein level, CXCR4 and CD133 were determined through immunoblotting and flow cytometry. CD133 was clearly detectable in OVCAR-3, OVCAR-4 and OVCAR-5, ovarian cell lines and in KM-12, Colo-205, HT-29, HCT-116 and SW620 colon cancer cell lines. CD133 was weakly expressed in SK-MEL28 and SK-MEL2, melanoma cell lines, while CD133 was not detectable in the remaining cell lines (Fig. 1A). Conversely, CXCR4 was detectable in the majority of the cancer cell lines (Fig. 1A). CD133 and CXCR4 surface level was detected through flow cytometry showing heterogeneous levels in the 60 cell lines. As expected, CXCR4 was highly expressed in leukemia cell lines (90% in CEM and 71% in MOLT-4) and in colon cancer cell line HT-29, breast cancer MCF-7 and ovarian OVCAR-4 cell lines. Interestingly, while CD133 protein was expressed in membrane, most of CXCR4 was not detectable in membrane in the epithelial cell lines (Fig. 1B).

### CXCR4^+^CD133^+^ ovarian cancer cells display stem cell properties

**Figure 2:**
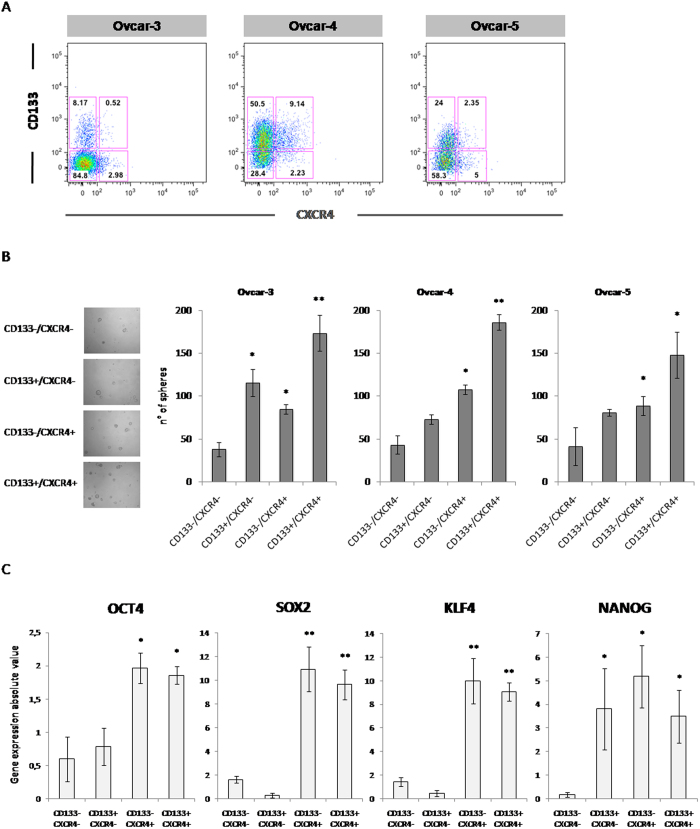
CXCR4^+^CD133^+^ ovarian cancer cells show stem cell properties. ** (A)** For *in vitro* and *in vivo* experiments, cells were double-stained for CD133 and CXCR4. Four distinct phenotypic subpopulations, specifically CD133^−^CXCR4^−^, CD133^−^CXCR4^+^, CD133^+^CXCR4^−^ and CD133^+^CXCR4^+^, were isolated. **(B)** Sphere formation capacity of sorted population in OVCAR-3, OVCAR-4 and OVCAR-5 cells. Tumor spheroids under non-differentiating and non-adherent conditions are known to contain a greater number of CSCs, images of OVCAR-5 sphere as representative of anchorage-independent growth, and tumor spheroid formation was reported. (Left side, B); Bar graph depicting number of spheres observed in sorted cells culture (Right side, B); QPCR analysis of pluripotency-associated genes (OCT4, SOX2, KLF4, NANOG) in OVCAR-5 sorted populations **(C)**. The data represent the mean ± SD. Asterisk (*) represents *p* values < 0.05; double asterisk (**) represents *p* values < 0.001.

Since CXCR4 and CD133 were previously reported as stem cell markers^27^,^28^, the OVCAR-3, OVCAR-4 and OVCAR-5 ovarian cancer cell lines sorted for CXCR4-CD133 were evaluated for cancer stem cell features (Fig. 2A). Through the sphere-forming assay, the sorted population CD133^+^CXCR4^+^ and the CD133^−^CXCR4^+^ demonstrated the highest efficiency in sphere formation in OVCAR-3, OVCAR-4 and OVCAR-5 cells (Fig. 2B). To further investigate stemness, the pluripotency associated markers OCT4, SOX2, KLF4 and NANOG were evaluated in OVCAR-5 sorted cells. CD133^+^CXCR4^+^ and CD133^−^CXCR4^+^ cells highly expressed SOX2, KLF4, NANOG compared with CD133^−^CXCR4^−^ and CD133^+^CXCR4^−^ cells (Fig. 2C). The most stringent stem cells feature is the tumor-forming assay; to this aim 1 × 10^3^ and 1 × 10^4^ OVCAR-5 and OVCAR-4 derived cells sorted as CD133^−^CXCR4^−^, CD133^+^CXCR4^−^, CD133^−^CXCR4^+^ and CD133^+^CXCR4^+^ were inoculated in nude mice. When 10,000 cells were subcutaneously inoculated in nude mice the number of implants developing tumor was 1/8 for CD133^−^CXCR4^−^, 2/8 for CD133^+^CXCR4^−^, 6/8 and 8/8 in cells respectively CD133^−^CXCR4^+^ and CD133^+^CXCR4^+^. When 1,000 cells were inoculated 0/8 for CD133^−^CXCR4^−^ formed tumor, 1/8 for CD133^+^CXCR4^−^, and 5/8 and 8/8 in cells respectively CD133^−^CXCR4^+^ and CD133^+^CXCR4^+^. Similar results were obtained using OVCAR-4 cells (Table 1).

### CXCR4^+^CD133^+^ ovarian cancer cells possess resistance to chemotherapy, migration and colony forming capabilities

**Figure 3:**
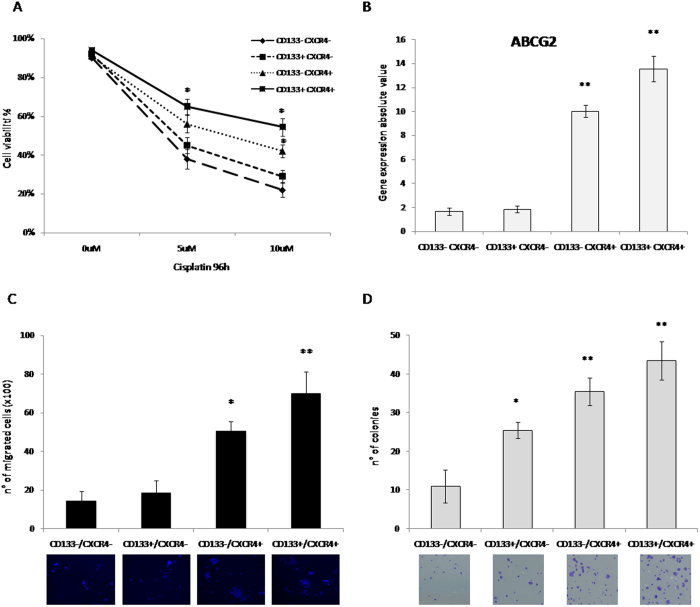
CXCR4^+^CD133^+^ ovarian cancer cells possess resistance to chemotherapy, migration and colony forming capabilities. **** OVCAR-5 sorted cells **(A)** Cytotoxicity assay in the presence of Cisplatin (0, 5, 10M). **(B)** QPCR analysis of ABCG2. **(C)** Migration assay toward CXCL12 and **(D)** Clonogenic assay. A representative photograph is provided in the lower panel. (*p < 0.05, **p values < 0.001, compared with the CD133-CXCR4- group).

Cancer stem cells generally display features of higher aggressiveness. Accordingly, CD133^+^CXCR4^+^ OVCAR5 sorted cells were evaluated for sensitivity to cisplatin, a commonly used agent for the treatment of ovarian cancer, and for the expression of ABCG2, a surface transporter associated with resistance to chemotherapy. OVCAR-5 CD133^+^CXCR4^+^ were less sensitive to cisplatin and expressed the highest level of ABCG2 transporters (Fig. 3A–B) displaying a drug resistant phenotype. To correlate the CXCR4/CD133 with functional CXCR4-CXCL12 axis, migration assay was performed in OVCAR-5 CD133^+^CXCR4^+^ sorted cells. CD133^+^CXCR4^+^ and CD133^−^CXCR4^+^ OVCAR-5 cells more efficiently migrated toward CXCL12. As expected CXCR4 regulates migration, but the concomitant expression of CXCR4 and CD133 further increases migratory capability (Fig. 3C). Moreover, colony formation capability was evaluated in OVCAR-5 sorted population. A higher number of clones was generated from CD133^+^CXCR4^+^ and CD133^−^CXCR4^+^ cells (35.5 ± 3.7 and 43.5 ± 4.2) compared to CD133^−^CXCR4^−^ and CD133^+^CXCR4^−^ (11 ± 4.7 and 25.5 ± 2.4) (Fig. 3D). Taken together these data demonstrate that CD133^+^CXCR4^+^ ovarian cancer cells display features of higher malignancy similar to what has been described for the cancer stem cell population.

### Cancer stem cell markers CXCR4, CD133, CD44, CD24 are heterogeneously expressed in human ovarian epithelial cancer

**Figure 4:**
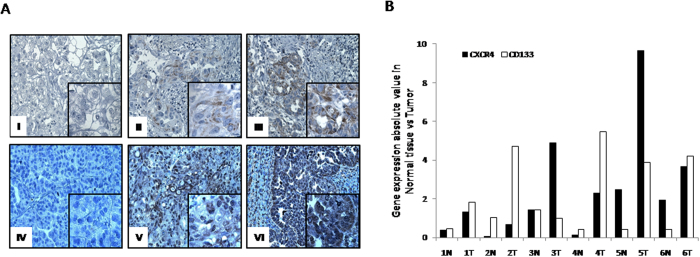
CXCR4 and CD133 are highly expressed in ovarian cancer patients. **** (**A**) Representative panel of immunohistochemistry for CD133 and CXCR4 expression (Magnification 200X; insert 400X). Non-homogeneous and focal cytoplasmic and membranous CD133 expression rated as negative (I), focally low (1–10% cancer cells) (II), focally high CD133 expression (>10% stained cancer cells) (III). Extensive homogeneous cytoplasmic and membranous, CXCR4 staining was reported rated as negative (IV) moderate (<50% of cancer cells) (V) and high expression (>50% of cancer cells) (VI). (**B**) QPCR analysis of CXCR4 and CD133 in freshly resected ovarian tumors and corresponding normal ovarian tissue.

Previous studies reported that CD133, CD24 and CD44, might identify cancer stem cells (CSCs) in ovarian or other solid tumors^29^,^30^,^31^. To verify the role of CXCR4 and CD133 in ovarian cancer patients, CXCR4, CD133, CD44, CD24 were evaluated in 37 surgically resected primary ovarian epithelial tumors (Table 2). Single-cell suspensions from dissociated tumor tissue and normal adjacent ovarian tissue were analyzed. The most striking evidence was that CD44, CD24, CD44/CD24 and CXCR4, CD133 and CXCR4/CD133 were detectable at extremely variable levels in primary tumors (Table S1 available in online Supplementary Material). CXCR4 expression was low (ranging from 0% to 62.5%, median 2.4%) and CD133 level was even lower (ranging from 0% to 48%, median 1.25%) in the fresh ovarian cancer cells. Concomitant expression of CXCR4^+^CD133^+^ was detected in 18 out of 37 tumors tested (ranging from 0% to 18%, median 0.07%). The relative expression of the putative stem cell markers were reciprocally and significantly associated (**Table S2**). Immunohistochemical CXCR4 and CD133 evaluation was also conducted (Fig. 4A). CXCR4 was highly expressed (>50% of tumors cells displaying stained) in 12/13 tumors (92.4%), moderately (<50% of tumors cells displaying stained) expressed in 1/13 (8.3%); CD133 was highly expressed (>20% of tumors cells stained) in 4/13 (38.5%), expressed at low levels in 8/13 (53.8%) and negative in 1/13 (8.3%). In addition higher mRNA for CD133 and CXCR4 was also detected in 6/37 primary ovarian tissues compared to surrounding unaffected tissues (Fig. 4B).

## Discussion

In this manuscript we took advantage of the NCI 60 cell lines to investigate the level of two putative cancer stem cell markers, CXCR4 and CD133. Screening revealed that, ovarian OVCAR-3, -4 and -5 and colon cancer HT-29, HCT-116 and SW620 over expressed both proteins OVCAR-5 CXCR4^+^CD133^+^ identified cells with cancer stem properties as demonstrated through *in vitro* spheres formation, clonogenic properties and stemness related genes expression (OCT-4, SOX2, KLF4, NANOG). Most strikingly, CXCR4^+^CD133^+^ OVCAR5 cells formed the highest number of tumors when inoculated in nude mice. Moreover, OVCAR-5 CXCR4^+^CD133^+^ cells migrated toward the CXCR4 ligand CXCL12, were less sensitive to the most popular chemotherapeutic agent utilized in ovarian cancer, cisplatin, and over expressed the drug resistance transporter ABCG2. Cancer cells overexpressing CXCR4 and CD133 were evaluated in fresh primary human ovarian cancer revealing extremely variable levels of CXCR4^+^/CD133^+^ and CD24^+^/CD44^+^ positive cells, without significant differences among the group of patients. Ovarian cancer is considered the most lethal gynaecological malignancy, accounting for one third of cancers occuring in women^32^. The recent cancer stem cell hypothesis suggests that ovarian cancer might be driven and sustained by a subset of cells with stem cell characteristics including unlimited proliferative potential and resistance to therapy^33^. Such cells could explain why cancers often relapse despite clinical remission with initial therapy. Over time, it would take only a few treatment-resistant stem cells to repopulate the tumor^28^,^34^,^35^. Epithelial ovarian cancers are known to occur in transitional zones between two types of epithelium, whereas others have been shown to originate in epithelial tissue stem cells. Recent evidence in mice focuses attention on the ovarian hilum region, the transitional area between the ovarian surface epithelium, mesothelium and tubal (oviductal) epithelium, defined as a previously unrecognized ovarian cancer stem cell niche. Cells of the hilum cycle slowly and express stem and/or progenitor cell markers ALDH1, LGR5, LEF1, CD133 and CK6B^36^. Ovarian cells with stemness properties have been identified in populations of CD44-positive^4^,^5^, CD133-positive^6^,^8^ cells, Hoechst-excluding cells (the side population)^9^, and aldehyde dehydrogenase (ALDH1A1)-positive cells^10^,^11^,^12^,^13^ and are associated with poor clinical outcome. With the intent to isolate CSC ovarian cancer, 37 primary tumor samples were evaluated for the expression of CXCR4/CD133 and CD44/CD24. Although no significant prognostic correlations were found among the 18 patients overexpressing CXCR4 and CD133, the majority of patients expressing CXCR4 and CD133 or CXCR4 displayed poor prognosis. Although CD133 was detected in a minimum percentage of OVCAR-5 cells (5.6% to 16.0%) it was recently shown that targeting a small number of CD133 positive cells can selectively decrease tumor progression of OVCAR-5-luc tumors^37^. In agreement with our results reported here, ALDH, stem cell markers, and CD133 were variably expressed resulting in absence of significant correlation assumable due to the low number of samples examined^35^. CD133 was significantly associated with high-grade serous carcinoma, late-stage disease, ascites severity and resistance to therapy when evaluated through Tissue Microarray in 400 ovarian carcinoma samples^38^. The main cause of treatment failure and death in ovarian cancer patients is uncontrolled invasion and metastasis^2^. The CXCR4-CXCL12 axis, that plays a central role in metastases dissemination, was previously described in ovarian cancer where the involvement of CXCR4-CXCL12 axis in collagen invasion and proliferation was relevant to the metastatic epithelial ovarian cancer^39^,^40^,^41^,^42^,^43^. In a study of expression of 14 chemokines receptors, only CXCR4 was expressed and functional within ovarian cancer cell lines^41^. CXCR4 antagonist such as AMD3100 are available as hematopoietic stem cell precursor mobilizer agents^44^ and other inhibitors are in clinical development^45^. We recently discovered a new class of CXCR4 antagonists^46^ that, alone or in association with chemotherapeutic agents and/or CD133 targeting agents, might reduce chemoresistance and development of secondary lesions.

The collection of data sets related to the NCI 60 cell lines provides an unparalleled public resource for integrated chemo genomic studies aimed at elucidating molecular targets, identifying biomarkers for personalization of therapy and understanding mechanisms of chemosensitivity and chemoresistance^47^. Although cell lines have been removed from their *in vivo* context and selected for growth in culture, and thus cannot be considered accurate surrogates for clinical tumors, they are reasonably stable and reproducible over extended time periods, available in large quantities, and manipulable. Here we demonstrated that screening a wide array of human cancer cells is a valid tool to identify relevant biological cell features.

CXCR4 and CD133 expression identified a discrete population with stem cell properties in human ovarian cancer cells that might be critical for tumor development and chemo resistance. This cell population represents a potential therapeutic target.

## Material and Methods

### Cell culture

The NCI 60 cancer cell line collection^6^ was obtained directly from the National Cancer Institute’s Developmental Therapeutics, program (NCI DTP) and maintained in RPMI 1640 media (Invitrogen), containing 10% Fetal Bovine Serum (ATCC).

### Cytotoxicity assay

The cells were seeded in 96-well plates (Nalgene Nunc International, Rochester, NY) at a concentration of 1 × 10^4^ cells/well in 100 μl of complete medium. The cells were allowed to adhere and 24 hours later were treated for 72 hours. Cytotoxic activity was measured by the sulforhodamine B (SRB) assay according to the manufacturer’s instructions. Cells were fixed in 200 μL of 10% trichloracetic acid (Sigma, St. Louis, MO), incubated for 1 hour at 4 °C. The cells were stained with 100 μL SRB (0.4%) (Sigma) for 15 minutes and resuspended in 10 mM unbuffered Tris. The absorbance at 540 nm was measured using an ELISA reader (Mithras LB 940, Berthold Technologies). The protein absorbance of the viable cells at each concentration was expressed as the relative percent absorbance compared with the control well without drug exposure. Each experiment was performed using three replicated wells at same drug concentrations and all the experiments were repeated three times.

### Flow cytometry

Adherent cancer cells at 50–70% confluent were detached with 2 mmol/L EDTA in PBS, washed, re-suspended in ice-cold PBS. Tumors were mechanically and enzymatically dissociated, cell pellets were collected by centrifugation, washed, passed through a 40 μm filter, then passed through a Standard Hub Pipetting needle, to form single-cell suspensions. The cells were incubated for 10 min at 4 °C with the following Anti-Human monoclonal antibodies: CD326 (EpCAM)-FITC, (130-080-301 Miltenyi Biotec), CD133/1-PE (clone AC133, Miltenyi Biotec, CA, USA), CD184/CXCR4 PE/Cy5 (306508 clone 12G5 BioLegend), CD45 APC-Cy™7 (557833 BD Pharmingen™), CD44 FITC (555478 BD Pharmingen™), CD24 PE (555428 BD Pharmingen™), CD45PE-Cy™5 (555484 BD Pharmingen™), CD34-PE (130-081-002 Miltenyi Biotec). Appropriate fluorochrome-conjugated, isotype matched antibodies were used as control to establish background staining. Samples were acquired on a FACS ARIA, and data were analyzed with DIVA software (BD, Biosciences, San Diego, CA). FACS was performed with >1 × 10^5^ cells using under low pressure in the absence of UV light.

### Western blot analysis

For the analysis of protein expression, the cells were homogenized in lysis buffer (40 mM Hepes pH 7.5, 120 mM NaCl, 5 mM MgCl_2_, 1 mM EGTA, 0.5 mM EDTA, 1% Triton X-100) containing protease (Complete Tablets-EDTA-free, Roche) and phosphatase (20 mM a-glycerolphosphate, 2.5 mM Na-pyrophosphate) inhibitors. Twenty-five micrograms of cell lysates were analyzed on 12%-10% SDS-PAGE and the following primary antibodies were used: anti-CXCR4 (Abcam, ab2074), anti-CD133 (Abcam, ab19898), anti-tubulin (Santa Cruz Biotech, CA). Anti-mouse and anti-rabbit IgG coupled to peroxidase were used as secondary antibodies (Santa Cruz Biotech, CA) and the signal was revealed through chemo luminescent detection kit (ECL detection kit, Amersham Biosciences, Freiburg, Germany).

### Sphere formation assay

Spheres were generated by culturing ~2 × 10^4^ ovarian cancer cells in suspension in serum-free DMEM/F12 supplemented with B27 (1:50, Invitrogen,), 20 ng/ml bFGF and 50 units/ml pen/strep for a total of 7 days, allowing spheres to reach a size of >75 μm.

### Cell migration assay

Migration was assessed in 24-well Transwell chambers (Corning Inc., Corning, NY) using inserts with an 8-μm pore membrane. Membranes were pre-coated with collagen (human collagen type I/III) and fibronectin (10 g/ml each). Test cells were placed in the upper chamber (2.0 × 10^5^ cells/well) in DMEM containing 0.5% BSA (migration media); cells migrate toward a medium containing CXCL12 (100 ng/ml) in the lower well. After 16 hours incubation, cells on the upper surface of the filter were removed using a cotton wool swab. The cells were counted in ten different fields (magnification 400 x).

### Clonogenic assay

About 5 × 10^2^ cells were added into each well of a six-well culture plate (three wells for each group). After incubation at 37 °C for 21 days, the cells were washed twice with PBS and stained with 0.1% crystal violet solution. The number of colonies containing ≥20 cells was counted under a microscope.

### *In vivo* tumorigenicity assays

Ovarian cancer cells OVCAR-5 were sorted for CXCR4 and CD133 as previously described^29^. For tumorigenicity assays, serial dilutions of single-cells re-suspended in Matrigel^TM^ (BD Bioscience, Heidelberg, Germany) were subcutaneously injected into female nude NMRI nu/nu mice (Janvier, Le Genest-Saint-Isle, France).

### Tissue collection

Thirty-seven (37) primary ovarian epithelial cancer tissue specimens were obtained from the Uro-Gynecological Department of Istituto Nazionale per lo Studio e la Cura dei Tumori, “Fondazione Giovanni Pascale” (Naples, Italy), between March 2009 and March 2011. All epithelial ovarian cancer were FIGO stage II - IV disease. Informed consent was obtained from all patients before tissue procurement. Patient management and follow up procedures were carried out in accordance with the particular protocols of the center.

### RNA preparation and qRT-PCR

Total RNAs from human primary ovarian cancer tissue and cells were extracted with TRIzol (Life Technologies,) according to the manufacturer’s instructions. 1μg of total RNA was reverse-transcribed with SuperScript II reverse transcriptase (Life Technologies) using random hexamers. Quantitative real-time PCR was performed with an Applied Biosystems 7500 real-time thermo cycler (Applied Biosystems,) using Fast SYBR Green (Qiagen) as the manufacturer’s instructions. The list of utilized primers is available in online Supplementary Material (Table S3).

### Immunohistochemical Analysis

Formalin-fixed and paraffin-embedded sections were subjected to high-temperature antigen retrieval and stained using Histostain-Plus Streptavidin-Peroxidase Detection kit (Life Technologies). Primary antibodies for IHC used were: Monoclonal Anti-Human CXCR4 (mab172, clone 44716, diluition 1:1000 R&D system), and Monoclonal Anti-human CD133/1 (clone AC133, diluition 1:100, Miltenyi Biotec, CA, USA,). Staining for CXCR4 and CD133 was categorized into semiquantitative classes based on the rate of stained (positive) tumour cells in 10 high power tumour field (400x)/slide: rated as negative moderate (<50% of cancer cells) and high expression (>50% of cancer cells) for CXCR4; rated as negative, focally low (<10% cancer cells) and focally high CD133 expression (>10% stained cancer cells). Semiquantitative classes were chosen by our pathologists after consensus discussion and careful revision of all slides. The distribution of the CXCR4 and CD133 protein was analyzed by live imaging using a Zeiss AxioScope light microscope.

### Statistical Analysis

Results for continuous variables are presented as means ± standard deviation unless stated otherwise, and significance was determined using the Mann-Whitney test. Statistical analysis was performed using the MedCalc Statistical Software version 9.3.7.0 (Microsoft, Inc., Belgium).The Spearman correlation test was used to evaluate the association between putative CSC markers expressions. The correlations between CSC markers and clinical pathologic features of patients were analyzed x^2^ test. P-values <0.05 were deemed statistically significant.

### Ethics statement

RNA and tissues samples from patients were obtained together with their informed consent in accordance with the Helsinki Declaration as revised in 2000. Mouse experiments were carried out in accordance with Italian Legislative Decree 116/1992 strictly comply with the European Guideline 86/609/EEC updated by Directive 2010/63. All experimental protocols were approved by Istituto Nazionale Tumori, “Fondazione G. Pascale” Ethics committee.

## Additional Information

**How to cite this article**: Cioffi, M. *et al.* Identification of a distinct population of CD133^+^CXCR4^+^ cancer stem cells in ovarian cancer. *Sci. Rep.* **5**, 10357; doi: 10.1038/srep10357 (2015).

## Supplementary Material

Supplementary Information

## Figures and Tables

**Table 1:** Limiting dilution tumor formation of sorted tumor cells.

**Cell type**	**number of injected cells and tumor formation**	
**Ovcar-5**	**1×10**^4^	**1×10**^3^
CD133^−^ CXCR4^−^	1/8	0/8
CD133^+^CXCR4^−^	1/8	1/8
CD133^−^ CXCR4^+^	6/8	5/8
CD133^+^CXCR4^+^	8/8	8/8
		
**Ovcar-4**		
CD133^−^ CXCR4^−^	2/8	1/8
CD133^+^CXCR4^−^	4/8	2/8
CD133^−^ CXCR4^+^	6/8	4/8
CD133^+^CXCR4^+^	8/8	7/8

**Table 2:** Clinical characteristics of patients.

	**No.**	**%**
**Age (years;<median/≥median)**		
<61	9	24,30
≥61	28	75,70
		
**Recurrence/Progression**		
No	8	21,60%
Yes	29	78,40%
		
**Grading**		
I	2	6,10%
II	1	3,00%
III	30	90,90%
		
**FIGO Stage**		
I - II	4	13,30%
III-IV	26	86,70%
		
**Histotype**		
Clear Cell	6	16,70%
Endometrioid	2	5,60%
Mucinous	1	2,80%
Serous	24	66,70%
Small Cell	1	2,80%
Undifferetiated	2	5,60%
		
**Survival status**		
AWD	9	25,00%
DOD	16	44,40%
NED	11	30,60%
		
**Treatment Response**		
NA	8	27,60%
PD	3	10,30%
CR	13	44,80%
PR	5	17,20%

AWD: Alive with Disease; DOD: Died of Disease; NED: No Evidence of (Active) Disease; PD: Progressive Disease; PR: Partial Response; CR: Complete Response; NA: Not Assessable.
