# Supplementary material for: Identification of a distinct population of CD133+CXCR4+ cancer stem cells in ovarian cancer
Source: Sci Rep. 2015 May 28;5:10357. doi: 10.1038/srep10357 (PMC4650662; doi:10.1038/srep10357)
Supplement: Supplementary Information [file srep10357-s1.pdf]

Supplementary Information

Identification of a distinct population of $CD133^{+}CXCR4^{+}$ cancer stem cell in ovarian cancer.

Michele Cioffi, Crescenzo D'Alterio, Rosalba Camerlingo, Virginia Tirino, Claudia Consales, Anna Riccio, Caterina Ieranò, Sabrina Chiara Cecere, Nunzia Simona Losito, Stefano Greggi, Sandro Pignata, Giuseppe Pirozzi, Stefania Scala.

Table S1. Patient characteristics and expression of putative CSC markers in primary human ovarian epithelial tumors samples analyzed by FACS

n	Age	FIGO stage	Grade	Histotype	Survival status*	Survival (months)	% CD44+	% CD24+	% CD44+CD24+	% CD133+	% CXCR4+	% CXCR4+CD133+
1	67	IIIC	3	Endometrioid	DOD	15	3,9	7,8	0,9	0,65	0,62	0,21
2	55	II	3	Serous	DOD	27	31	4,6	4,3	0,02	0,13	0
3	57	IIIC	3	Clear	NED	35	0,7	26,7	0,2	5,35	7,66	3,95
4	61	IIIA	3	Clear	AWD	33	23,9	23,1	13,2	47,88	11,33	5,78
5	48	IIIC	3	Clear	DOD	38	3,7	0,4	0,2	0,9	1,02	0,04
6	45	IIIC	3	Serous	NED	35	6,6	10,9	5,6	10,97	3,79	1,52
7	47	IIIC	3	Anaplastic	AWD	37	27,2	2,9	15	0,9	12	0
8	57	IIIC	3	Serous	NED	31	79,6	37,2	24	10	20,3	2,9
9	57	IIIC	3	Serous	AWD	31	58,9	41,3	50,1	9	1,5	0
10	48	IIIC	3	Serous	DOD	24	21	20	10	3	26,6	2,6
11	85	IIC	3	Serous	NED	27	26	1,3	15	0	2,4	0
12	24	IC	NA	Serous	AWD	30	0,2	1	0	0	20	0,2
13	61	IV	2	Serous	NED	33	42,2	23	21,5	1,3	0,3	0
14	42	IIIC	3	Serous	DOD	6	2,55	4,7	0,1	1,3	0,87	0,07
15	71	IIIC	3	Serous	DOD	41	39,3	1,23	0,89	0,48	0,25	0
16	51	IIIC	3	Mucinous	NED	45	1,81	1,93	0,04	3,52	10	0,04
17	45	IIIC	3	Serous	AWD	45	36,8	22,11	14,18	2,62	0,31	0,12
18	65	IIIC	3	Serous	DOD	37	32,32	2,22	1,19	1,89	4,53	0,74
19	71	IIIC	3	Serous	DOD	11	8,98	20,59	4,78	2,25	6,79	1,04
20	62	IIIC	3	Serous	AWD	40	8,38	11,24	3,4	1,25	0,5	0,32
21	73	IIIC	3	Serous	DOD	71	33,32	1,44	1,02	0,57	0,26	0,02
22	32	IIIA	3	Clear	AWD	98	17,34	2,49	0,71	7,31	4,72	0,53
23	62	IIIC	3	Endometrioid	NED	49	33,32	66,55	1,72	21	39,21	18
24	47	IIIC	1	serous	NED	34	2,6	53,6	1,25	8,25	62,52	6,72
25	51	IIIA	3	Clear	NED	45	6,89	55,47	3,37	5,74	23,88	4,2
26	66	IIIC	3	Clear	DOD	30	6,89	9,13	1,2	0,4	30,61	0,07
27	62	IV	3	undiff	DOD	12	36,84	67,39	28,28	3,22	11,24	1,42
28	49	IIIC	3	Endometrioid	DOD	3	4,48	46,75	0,75	1,16	0,7	0
29	57	NA	NA	NA	DOD	14	7,1	20,7	3,3	2,7	2,6	0,3
30	71	NA	3	Serous	NA	1	12,3	8,9	2,2	0,3	0	0
31	71	NA	NA	Serous	DOD	51	89	0	0,6	0,8	0	0
32	73	IV	3	Serous	AWD	41	22	0,3	0	0	1,1	0
33	62	NA	NA	Serous	NED	30	43,4	34,7	23,9	0,3	0	0
34	62	NA	3	Serous	DOD	8	3,2	27,8	1,1	0	4,5	0
35	68	IIA	3	Clear	NED	52	31,12	3,5	3,3	1,3	0	0
36	68	NA	1	Serous	AWD	42	2,85	2	0,31	0,2	0	0
37	71	NA	3	Serous	DOD	38	37,7	38,5	12,6	0,3	0	0,7

*NED , No evidence of disease; AWD, alive with disease; DOD, died of disease.

Table S2. Correlation Analysis between Biomarkers using Spearman's rank correlation analysis

Variable	Rho	P value
**CXCR4 and CD133**	0,413	0,01313
**CXCR4 and CD44**	-0,37	0,02639
**CD133 and CD24**	0,482	0,003847
**CD24 and CD133**	0,482	0,003847
**CD24 and CD133+CXCR4+**	0,47	0,004803

Table S3. List of utilized primer sequences

Gene	Primer sense	Primer antisense
Nanog	tgaacctcagctacaaacaggtg	aactgcatgcaggactgcagag
Klf4	acccacacaggtgagaaacc	atgtgtaaggcgaggtggtc
Sox2	agaaccccaagatgcacaac	cggggccggtatttataatc
Oct3/4	cttgctgcagaagtgggtggaggaa	ctgcagtgtgggtttcgggca
CXCR4	ggtggtctatgttggcgtct	tggagtgtgacagcttggag
CD133	ggacccattggcattctc	caggacacagcatagaataatc
ABCG2	tcatgttaggattgaagccaaaggc	tgtgagattgaccaacagacctga
B-ACTIN	gcgagcacagagcctcgcctt	catcatccatggtgagctggcgg
